# Geographical distribution and regional differences in 532 clinical isolates of rapidly growing mycobacterial species in Japan

**DOI:** 10.1038/s41598-021-84537-7

**Published:** 2021-03-02

**Authors:** Keisuke Kamada, Atsushi Yoshida, Shigekazu Iguchi, Yuko Arai, Yutaka Uzawa, Satoshi Konno, Masahiro Shimojima, Ken Kikuchi

**Affiliations:** 1 Department of Infectious Diseases, Tokyo Women’s Medical University, 8-1 Kawada-Cho, Shinjuku-ku, Tokyo, 162-8666 Japan; 2 Department of Respiratory Medicine, Faculty of Medicine and Graduate School of Medicine, Hokkaido University, Kita 14-jo Nishi 5-chome, Kita-ku, Sapporo-shi, Hokkaido, 001-0014 Japan; 3 BML, Inc., Matoba 1361-1, Kawagoe-shi, Saitama, 350-1101 Japan

**Keywords:** Microbiology, Climate sciences, Environmental sciences

## Abstract

Infectious diseases caused by nontuberculous mycobacteria (NTM) are increasingly becoming a major global problem. Additionally, *Mycobacteroides abscessus* subsp. *abscessus* (MAB) infections are refractory to macrolides. This study was conducted to investigate the epidemiology of rapidly growing mycobacteria (RGM) species isolated from clinical specimens in Japan and assess differences in the regional distribution of lower respiratory specimens (LRS)- and non-lower respiratory specimens (NLRS)-derived species. 532 strains (427 LRS, 92 NLRS and 15 unknown specimens) were isolated in nine areas of Japan. We collected 418 specimens from Bio Medical Laboratories (BML), Inc., and 114 specimens from 45 hospitals in Japan. Their epidemiological differences were examined according to the specimen type, region, and climate. Fifteen species were identified. The proportion of *M. abscessus* group (MAG) strains was significantly lower in NLRS than in LRS (35.9% vs. 68.4%). The proportion of MAG strains was higher in northern Japan than in other regions (83.7% vs. 60.5%). Variations in strain abundance among RGM species was evident in regions with a mean annual temperature below 15 °C. We conclude that the proportions of MAG strains differed between NLRS and LRS in Japan. In addition, the mean annual temperature likely influenced the distribution of RGM species.

## Introduction

Infectious diseases caused by nontuberculous mycobacteria (NTM) are increasingly becoming a major global problem. Various NTM species exhibit regional epidemiological differences^1^. A recent large-scale questionnaire survey in Japan revealed an increased prevalence of NTM infections, with a fivefold increase in pulmonary infections caused by the *Mycobacteroides abscessus* group (MAG), a type of rapidly growing mycobacteria (RGM), over the past 15 years^2^. Among MAG infections, *M. abscessus* subsp. *abscessus* (MAB) infection is more refractory to macrolides than *M. abscessus* subsp. *massiliense* (MMA) infections^3,4^; however, strains with the C28 *erm*(41) sequevar often retain macrolide susceptibility^5^. Epidemiological analysis of NTM isolated from lower respiratory tract specimens (LRS) from three major commercial laboratories in Japan showed that the frequency of *M. abscessus* isolation was highest in the Kyushu-Okinawa area located in southern Japan^6^. Past NTM epidemiological studies suggested that climate conditions, such as temperature, precipitation, and water vapor pressure, affect NTM activity^7,8^.

Regional differences in NTM isolates have been extensively reported^8–10^. However, few studies have used genetic methods to identify NTM species. Furthermore, few reports have described the epidemiological characteristics of the region with respect to RGM alone^11^. Therefore, in this study, we investigated the epidemiology of RGM species isolated from clinical specimens in Japan and the differences in the regional distribution of LRS- and non-lower respiratory specimens (NLRS)-derived species. We performed polymerase chain reaction (PCR)-based typing of MAG to enable more detailed subspecies determination than previously achieved^12^ and confirmed the presence of the *erm*(41) sequevar type, which affects macrolide susceptibility among the isolated MAB strains. We also assessed regional differences in the isolated species and analyzed the relationships between strains of the major RGM species and climatic conditions of the prefectures from which the isolates were obtained.

## Results

**Figure 1:**
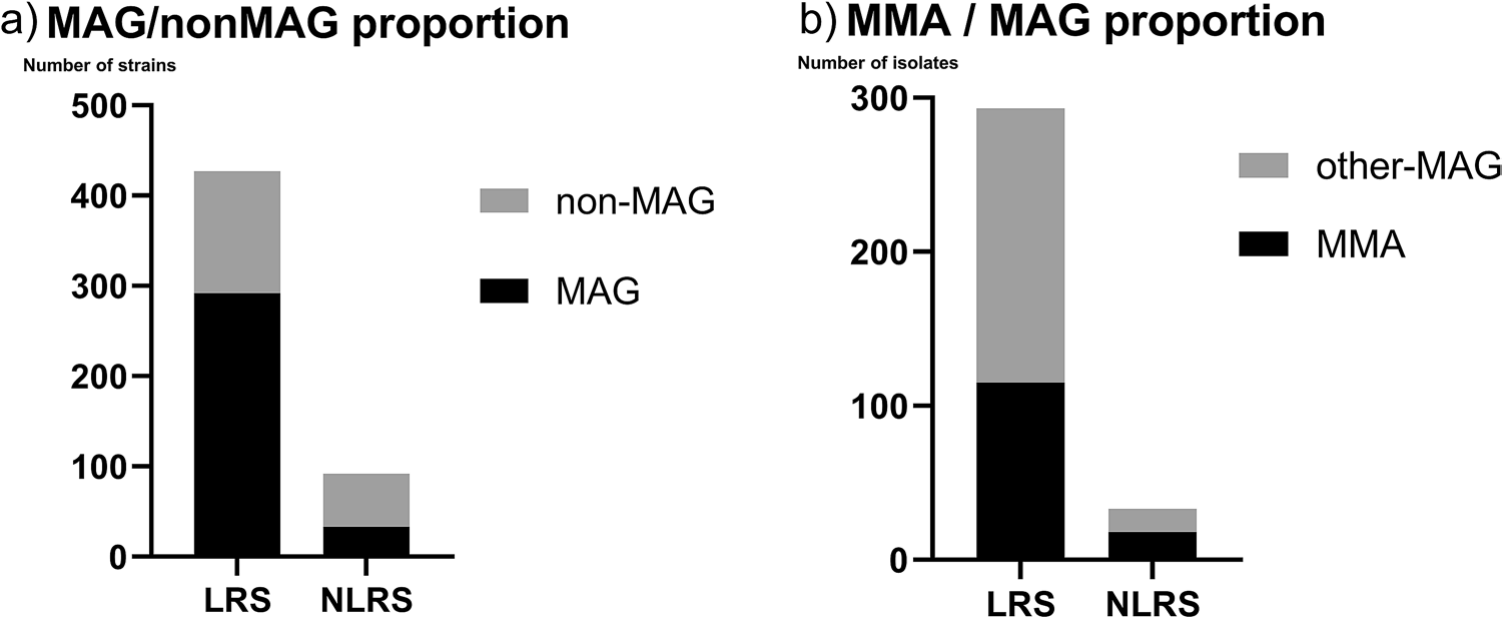
Proportion of *Mycobacterium abscessus* group (MAG) strains and subspecies separated for each sample type. (**a**) Non-MAG strains: rapidly growing mycobacteria species other than MAG; (**b**) non-*M. abscessus* subsp. *massiliense* (MMA) strains: subsp *abscessus*, subsp. *bolletii* and unclassified MAG strains.

Table 1 shows the list of identified RGM species for each specimen type. Fifteen RGM species were identified, with the most common MAG accounting for approximately 60% of the total RGM strains. The top three species isolated from LRS and NLRS were the same; however, the relative proportion of MAG was significantly lower in NLRS (35.9%, 33/92) than in LRS (68.4%, 292/427; *P* < 0.001; OR 0.26; 95% confidence interval [CI] 0.16–0.42; Fig. 1a). In addition, NLRS comprised a higher fraction of MMA (54.6%, 18/33) than LRS (39.3%, 115/293; *P* = 0.096; odds ratio (OR) 1.86; 95% CI 0.93–3.84) (Fig. 1b).

**Table 1:** Distribution of rapidly growing mycobacteria species by specimen type.

Pathogens	Total	Number of strains									Unkown
		LRS^A^	NLRS total	NLRS							
				Skin soft tissue	Abscess	PD-related	Blood	Otrrhea	Bone	Other	
**MAG**											
subsp. *abscessus*	190	170	15	2	4	3	1	2	2	1	5
subsp. *massiliense*	138	115	18	6	2	2	4	4			5
subsp. *bolletii*	4	4	0								
Unclassified	3	3	0								
*M. fortuitum*	91	71	20	9	4	3	1		1	2	
*M. chelonae*	60	39	20	12	3	1	2			2	1
*M. peregrinum*	11	11	0								
*M. mageritense*	10	1	8	3	1	2	1			1	1
*M. septicum*	5	4	1				1				
*M. mucogenicum*	4	1	2		1		1				1
*M. porcinum*	3	2	1		1						
*M. wolinskyi*	3	0	3		2		1				
*M. goodii*	2	1	1							1	
*M. iranicum*	2	1	1			1					
*M. senegalence*	3	3	0								
*M. canariasense*	1	0	1				1				
*M. immunogenum*	1	0	1		1						
*M. sphagni*	1	1	0								
Total	532	427	92	33	18	12	13	6	3	7	13

*PD* peritoneal dialysis.

^A^LRS contained 423 sputum and 4 bronchoalveolar lavage fluid samples.

Five RGM species, *M. mageritense*, *M. mucogenicum*, *M. wolinskyi*, *M. canariasense*, and *M. immunogenum*, were isolated mainly from NLRS. Three RGM species, *M. peregrinum*, *M. senegalense*, and *M. sphagni*, were isolated only from LRS. Only four RGM species, MAG, *M. fortuitum*, *M. chelonae*, and *M. mageritense*, were isolated from the skin and soft tissues and peritoneal dialysis-related specimens, whereas eight RGM species were isolated from abscesses and blood specimens.

**Figure 2:**
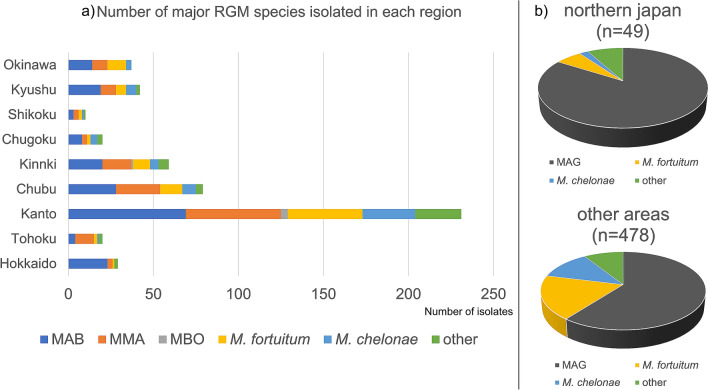
Major rapidly growing mycobacteria species isolated in each region of Japan. Strains isolated from both lower respiratory specimens (LRS) and non-lower respiratory specimens (NLRS) are included. (**a**) Each region of Japan; (**b**) North of Japan (Hokkaido and Tohoku) and other region.

Figure 2a shows the regional distribution of isolated RGM species. Most strains derived from NLRS were isolated in Kanto. The proportion of MAG was 89.7% (26/29) in Hokkaido, 75.0% (15/20) in Tohoku, 55.8% (129/231) in Kanto, 68.4% (54/79) in Chubu, 64.4% (38/59) in Kinki, 55.0% (11/20) in Chugoku, 60.0% (6/10) in Shikoku, and 66.7% (28/42) in Kyushu, and 62.2% (23/37) in Okinawa. The MAG proportion was higher in northern Japan (Hokkaido + Tohoku) than in other regions (83.7% vs. 60.5%; *P* = 0.001; OR 3.35; 95% CI 1.56–7.07; Fig. 2b). The same result was obtained even when the analysis was limited to LRS-derived strains (87.2% vs. 66.1%; *P* = 0.003; OR 3.51; 95% CI 1.50–7.92).

*Erm*(41) sequences were obtained from 186 MAB strains. Four strains could not be typed by PCR-direct sequencing. The overall proportion of MAB harboring the *erm*(41) C28 sequevar, considered to have good macrolide susceptibility, was 11.8% (22/186): 0% (0/23) in Hokkaido, 50% (2/4) in Tohoku, 7.2% (5/69) in Kanto, 10.7% (3/28) in Chubu, 10.0% (2/20) in Kinki, 12.5% (1/8) in Chugoku, 0% (0/2) in Shikoku, 5.2% (1/19) in Kyushu, and 61.5% (8/13) in Okinawa. The proportion of the C28 sequevar was significantly higher in Okinawa than in other areas (61.5% vs. 8.1%; *P* < 0.001; OR 18.06; 95% CI 5.60–56.6).

**Figure 3:**
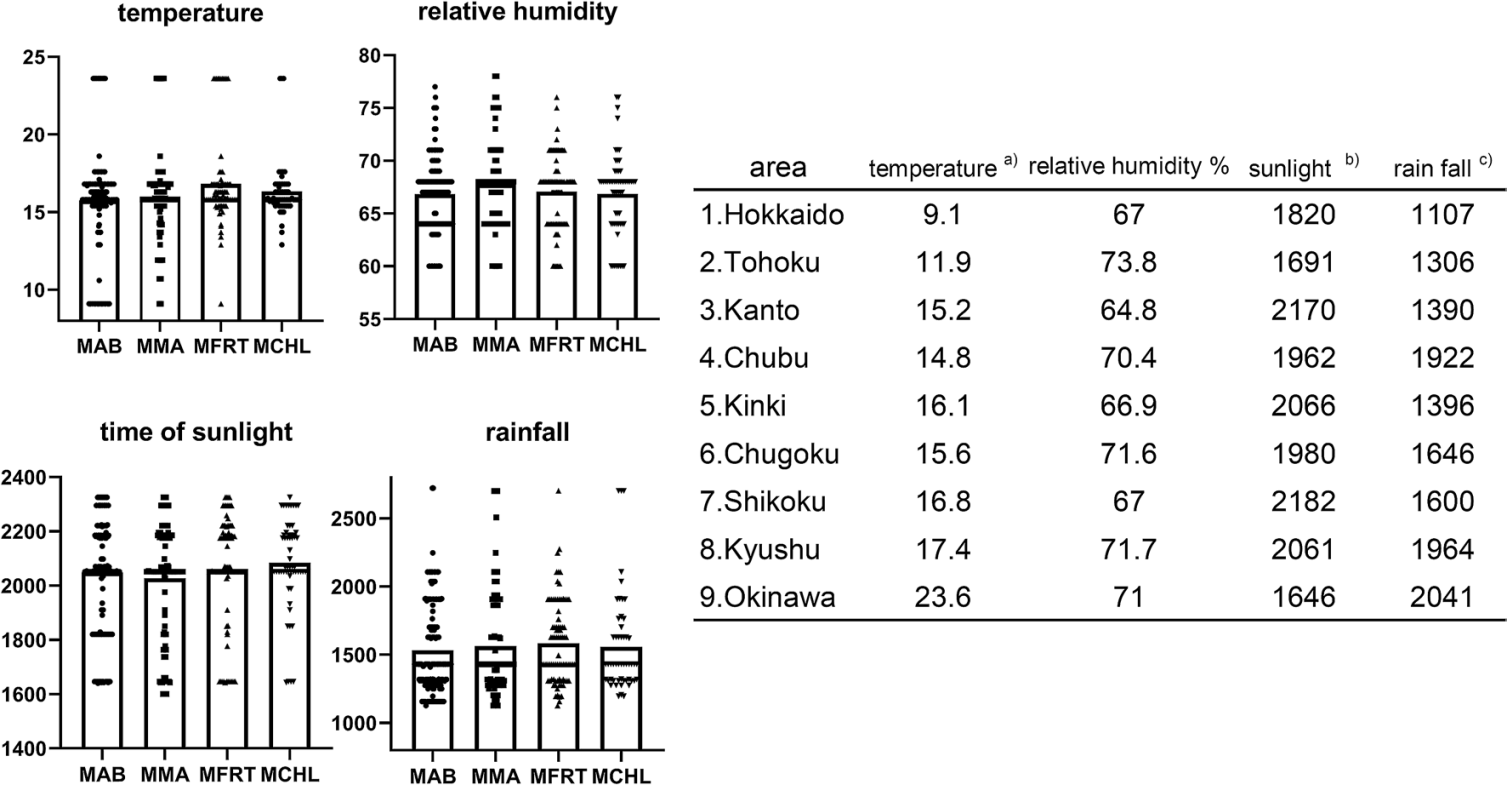
Weather conditions of the location where major rapidly growing mycobacteria species were isolated. (**a**) Temperature = average annual temperature, °C; (**b**) time of sunlight: h/year; (**c**) rainfall: mm/year. *MFRT M. fortuitum*, *MCHL M. chelonae.*

Figure 3 shows a plot of the weather conditions (annual average temperature, annual sunshine hours, annual rainfall and relative humidity) of the prefectures where each of the MAB, MMA, *M. fortuitum* and *M. chelonae* strains was isolated. There was no significant effect of all climate parameters (mean annual temperature, annual sunshine hours, annual rainfall, or relative humidity) on the distribution of RGM species (Kruskal–Wallis test with post-hoc test). However, the proportions of strains isolated in areas with an average annual temperature of 15 °C or less were as follows: MAB, 18.6% (35/188); MMA, 19.7% (27/137); *M. fortuitum*, 9.9% (9/91), and *M. chelonae*, 6.7% (4/60). The percentage of strains isolated in areas where the mean annual temperature was below 15 °C significantly differed between each RGM species based on the chi-squared test (χ^2^ = 8.87, *P* = 0.03). In 21 of the 47 prefectures from Japan, the annual average temperature was ≤ 15 °C. This suggests that the mean annual temperature influences the composition of RGM species isolated from each region.

## Discussion

This study was performed to clarify the epidemiology of RGM isolated from various types of clinical specimens. We conducted accurate species identification of RGM using detailed genetic methods and revealed valuable epidemiological information. Three new important results were obtained. First, the top three RGM species were the same for LRS and NLRS, although NLRS contained a significantly lower proportion of MAG strains. These results are consistent with previous data on the top four RGM species from LRS obtained by surveillance of major laboratories in Japan^6^. In addition, our findings agree with those in recent reports from Japan suggesting that within MAG, the MAB strains were isolated more frequently than the MMA strains^12^. However, few epidemiological RGM studies have examined NLRS. A study from the United States reported that 52 of 108 (48.1%) RGM strains obtained from NLRS were MAG^13^, but MAG subspecies were not investigated in the study. Additionally, there are no previous reports comparing MAG subspecies isolated from LRS and NLRS in the same region. We showed that MAB strains are dominant in LRS and MMA strains are dominant in NLRS isolated in Japan.

The second important result was that RGM strains isolated in northern Japan, a region with lower temperatures, fewer sunshine hours, and less precipitation, comprised a higher proportion of MAG strains than those in other regions. The proportion of MAG among the RGM isolated in northern Japan was higher than that from other regions (Fig. 2b). We examined several climatic conditions and found that the proportion of MAG was higher in regions where the average annual temperature was below 15 °C (21/47 prefectures). Of the 21 prefectures, 7 were in northern Japan (Hokkaido + Tohoku), 5 in Kanto, 5 in Chubu, 2 in Kinki, and 2 in Chugoku. The mean annual temperature in northern Japan was clearly lower than that in other regions (Hokkaido 9.1 °C, Tohoku 11.9 °C) (Fig. 3). This may have contributed to the high rate of MAG isolation. This may be because most specimens collected from northern Japan were obtained from LRS. However, the same result was obtained even when only LRS samples were compared (i.e., excluding samples obtained from NLRS). There are several reports that have investigated the relationship between RGM activity and climatic conditions^14,15^. In Houston, it was reported that RGM infections might increase in summer and fall, when temperatures are above 15 °C and the rainfall increases. About 90% of the RGM infections were caused by *Mycobacterium abscessus*, *M. fortuitum* complex, and *M. mucogenicum*^14^. In addition, surveillance in Queensland, Australia, suggested that temperature and rainfall may affect the incidence of NTM infections involving RGM, but there may be differences in the impact on different NTM species and regions^15^. It is difficult to determine the effect of rainfall on RGM activity, because increased rainfall suppresses aerosol dispersion of RGM-containing dust, but increases the aerosol dispersion when accompanied by heavy storms and high humidity, such as in cyclones^15^. In the earlier studies, seasonal variations within the same region was observed, however in this study, we compared the effects of climatic conditions on the proportion of RGM species isolated from different regions. We found that the average annual temperature may affect the composition of RGM species isolated in the area.

In an earlier study, the prevalence of pulmonary infections caused by MAG in a population was reported to be higher in southern Japan^6^; however, our survey cannot be presented in the form of prevalence per population, because the sampling is not complete, and it compares the proportion of MAG in the total isolated RGM for each region. Our survey is on a smaller scale compared to the one in the earlier report^6^; however, our method of species identification using genetic methods is more accurate. In addition, NLRS was not included in the earlier survey^6^. *M. fortuitum* and *M. chelonae* appear to be less active than MAG in areas where the mean annual temperature is below 15 °C (Fig. 3), which may result in the MAGs accounting for a higher proportion of the total. However, in future studies, this hypothesis needs further investigation with improved sampling bias.

The third important result was that the proportion of the C28 *erm*(41) sequevar among MAB strains was significantly higher in Okinawa than in other regions. Past global reports indicated that the C28 sequevar accounted for approximately 10–20% of MAB strains^16,17^, whereas in Japan, it occurred at a slightly low rate of 9% (ref.^12^) or 4% (ref.^18^). Our survey showed a slightly larger overall value, but it was substantially higher in Okinawa, where more than 60% of strains had the C28 sequevar. Interestingly, MAB isolated from skin and soft tissue infections in Taiwan was reported to have a high C28 prevalence of 36% (ref.^19^). Geographically, strains isolated in Okinawa may be more genetically similar to strains isolated in Taiwan than those of other Japanese areas may.

Our study had several limitations. In Japan, there is no biobank/central registry for RGM. We did not obtain strains from all clinical laboratories in Japan, which may have caused sampling bias. However, BML is one of the leading clinical laboratories in Japan, with 99 offices (10 in Hokkaido, 12 in Tohoku, 20 in Kanto, 18 in Chubu, 11 in Kinki, 8 in Chugoku, 7 in Shikoku, 11 in Kyushu, and 2 in Okinawa), so sampling bias might not be significant. There are two possible reasons for the increase in the number of strains in Kanto. The first reason is that the population of the Kanto region is about 40 million, which is clearly much larger than that of other regions and accounts for about one third of the total population of Japan. The second reason is that the author’s institution is located in the Kanto region, and many of the institutions (31/45) that requested strain analysis tended to be located nearby. So most NLRS-derived strains were isolated in Kanto and, therefore, may not reflect the epidemiology in Japan overall. Particularly, few NLRS-derived strains were obtained from northern Japan. It is unclear whether there were actually fewer cases of RGM extrapulmonary infections in northern Japan or whether our strain collection was biased. Because the number of MAB strains isolated from Okinawa was small, it is necessary to repeat the study with a larger number of strains from this prefecture. A further limitation is the lack of clinical correlation in this analysis. Despite these limitations, it is important to accurately identify RGM species by combining multiple genetic methods and show epidemiological characteristics of RGM by region and specimen type.

In conclusion, we conducted the first major epidemiological study of RGM in Japan. We showed that MAG accounted for a lower percentage of the strains isolated from NLRS compared to the fraction in LRS strains. The high rate of MAG isolation in northern Japan is likely to be related to the lower annual average temperature. The percentage of the C28 *erm*(41) sequevar in MAB was clearly higher in Okinawa than in other regions. Considering past NTM epidemiological studies, RGM epidemiology is likely to change over time. Therefore, it is necessary to continue collecting strains and carefully observing whether the proportions of RGM species identified in each region has changed.

## Methods

### Study design

**Figure 4:**
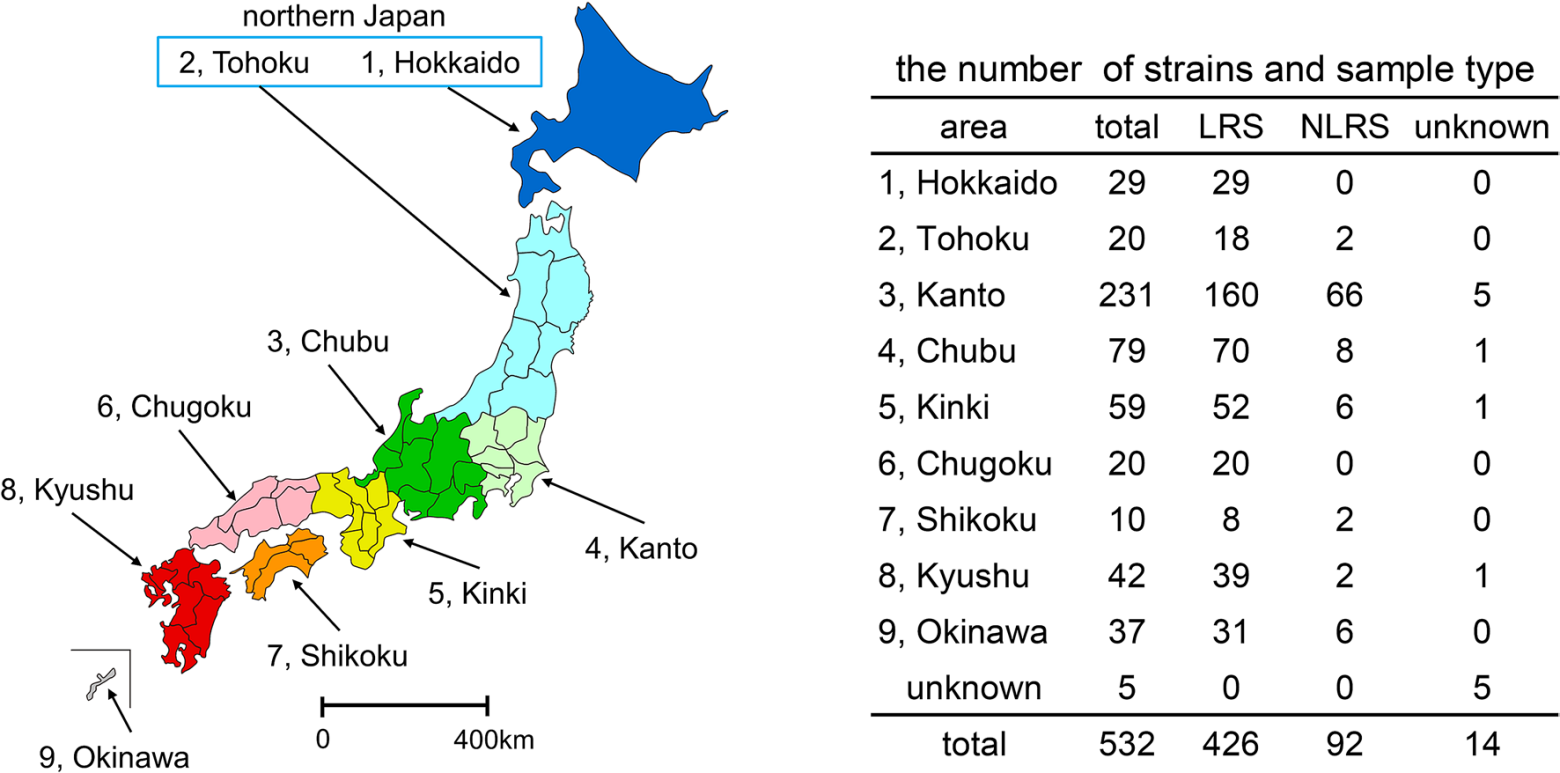
Distribution of rapidly growing mycobacteria (RGM) strains in different regions of Japan. Japan was divided into nine regions. Hokkaido and Tohoku were defined as Northern Japan. This map was created by Keisuke Kamada at the following site (https://n.freemap.jp/). The copyright of the completed map is held by Keisuke Kamada.

We investigated 532 RGM strains isolated from 427 LRS (423 sputum and 4 bronchoalveolar lavage fluid), 92 NLRS (33 skin and soft tissue specimens, 18 abscess specimens, 12 specimens related to peritoneal dialysis, 13 blood specimens, 6 otorrhea specimens, 3 bone specimens, 7 other specimens), and 15 unknown specimens obtained from human clinical samples in Japan from January 2012 to March 2019. Among the 532 specimens, 418 strains (395 LRS, 18 NLRS, and 5 unknown) were isolated at Bio Medical Laboratories (BML), Inc., one of the major clinical laboratories, and 114 (32 LRS, 74 NLRS, and 8 unknown) were isolated at 45 hospitals, in Japan. The clinical information was not available for the strains isolated in BML; however, the diagnosis of infection due to RGM was confirmed for the remaining 114 strains. Isolates were included if they fulfilled the following three criteria: (i) the culture was positive within 7 days after plating; (ii) the presence of mycobacteria was confirmed by smear examination, and (iii) the culture was free of other isolates from the same patient. We divided the 47 prefectures of Japan into nine areas (Hokkaido, Tohoku, Kanto, Chubu, Kinki, Shikoku, Chugoku, Kyushu, and Okinawa) and investigated whether there were regional differences in the RGM species. Figure 4 shows the number of strains and sample types per area.

### DNA extraction and PCR

All strains were subcultured on trypticase soy agar supplemented with 5% sheep blood at 35 °C (Nippon–Becton–Dickinson, Fukushima, Japan). DNA was extracted using an ISOPLANT II kit according to the manufacturer’s instructions (Nippon GENE, Tokyo, Japan). We identified RGM species by gene sequencing analysis of three housekeeping genes encoding RNA polymerase beta subunit (*rpoB*)^20^, heat shock protein 65 (*hsp65*)^21^, and superoxide dismutase A (*sodA*)^22^*.* MAG subspecies were determined by a PCR-based typing scheme^23^, and the sequevar type, including *erm*(41) C28, was confirmed by *erm*(41) sequencing analysis^24^*.* The list of primers used in these analyses is provided in Supplementary Table S1.

### Gene sequencing analysis and phylogenetic tree

The sequences were compared to those of typical strains. We constructed a phylogenetic tree to identify RGM species using the neighbor-joining method with Kimura’s two-parameter correction model (1000 bootstrap replications). We performed multisequence alignment using the genetic information processing software GENETYX ver.13 (GENETYX, Tokyo, Japan).

### Climatic conditions

The correlations of climate data from 47 prefectures (annual average temperature, annual rainfall, relative humidity, annual sunshine hours) with the presence of the major RGM species (MAB, MMA, *M. fortuitum*, and *M. chelonae*) were assessed. Climate data were imported from the Statistics Bureau of Japan in 2019^25^.

### Statistical analysis

Fisher's exact test was used for the following comparisons: 1. Proportion of MAG among the RGM isolated in LRS and NLRS. 2. Proportion of MMA among the MAG isolated from LRS and NLRS. 3. Proportion of MAG among the RGM isolates from northern Japan and other regions. 4. Proportion of the *erm*(41) gene of the MAB isolates with C28 sequevar between Okinawa and other regions.

The climatic conditions (mean annual temperature, rainfall, sunshine hours, and relative humidity) were compared for isolates of MAB, MMA, *M. fortuitum*, and *M. chelonae* using the Kruskal–Wallis test with post-hoc test.

The effect of the mean annual temperature on the different species was compared using the χ^2^ test for the proportion of strains isolated in areas with > 15 °C and < 15 °C. All statistical analyses were conducted with a significance level of α = 0.05 (*P* < 0.05) using GraphPad Prism ver. 8.2.0 for Windows (GraphPad Software, San Diego, CA, USA).

## Supplementary Information

Supplementary Table S1.

## Data Availability

The dataset generated and analyzed during the current study is available from the corresponding author on reasonable request.
